# Differences in Nutrient Requirements Imply a Non-Linear Emergence of Leaders in Animal Groups

**DOI:** 10.1371/journal.pcbi.1000917

**Published:** 2010-09-02

**Authors:** Cédric Sueur, Jean-Louis Deneubourg, Odile Petit, Iain D. Couzin

**Affiliations:** 1 Unit of Social Ecology, The Free University of Brussels, Brussels, Belgium; 2 Ethologie des Primates, Department of Ecology, Physiology, and Ethology, Institut Pluridisciplinaire Hubert Curien, Strasbourg, France; 3 Department of Ecology and Evolutionary Biology, Princeton University, Princeton, New Jersey, United States of America; McMaster University, Canada

## Abstract

Collective decision making and especially leadership in groups are among the most studied topics in natural, social, and political sciences. Previous studies have shown that some individuals are more likely to be leaders because of their social power or the pertinent information they possess. One challenge for all group members, however, is to satisfy their needs. In many situations, we do not yet know how individuals within groups distribute leadership decisions between themselves in order to satisfy time-varying individual requirements. To gain insight into this problem, we build a dynamic model where group members have to satisfy different needs but are not aware of each other's needs. Data about needs of animals come from real data observed in macaques. Several studies showed that a collective movement may be initiated by a single individual. This individual may be the dominant one, the oldest one, but also the one having the highest physiological needs. In our model, the individual with the lowest reserve initiates movements and decides for all its conspecifics. This simple rule leads to a viable decision-making system where all individuals may lead the group at one moment and thus suit their requirements. However, a single individual becomes the leader in 38% to 95% of cases and the leadership is unequally (according to an exponential law) distributed according to the heterogeneity of needs in the group. The results showed that this non-linearity emerges when one group member reaches physiological requirements, mainly the nutrient ones – protein, energy and water depending on weight - superior to those of its conspecifics. This amplification may explain why some leaders could appear in animal groups without any despotism, complex signalling, or developed cognitive ability.

## Author Summary

Making decisions together to reach a consensus is one of the most important challenges of any society. In some communities, however, some leaders have more weight in the decisions than the other individuals. Similar rules exist in animal societies. Studies on animal groups have shown that some individuals are more likely to be leaders because of their social power or the pertinent information they possess. This leader may be the dominant one, the oldest one, but also the one having the highest physiological need. However, how may other group members have their needs satisfied if always the same individual decides? To gain insight into this problem, we build an agent-based model where group members have to satisfy different needs but the individual with the lowest reserve decides when and where to move for all its conspecifics. This simple rule leads to a viable decision-making system that satisfies all individuals and suits their requirements. However, a single individual, the one with the highest needs, becomes the leader in 38% to 95% of cases according to the heterogeneity of needs in the group.

## Introduction

Social animals have to coordinate their activities in order to maintain the advantages of group living [1]–[3]. This coordination constitutes one of the major challenges of any animal society, including human beings, and arouses the interest of scientists, sociologists, and politicians [4]–[9]. Whatever the group size or the level of communication – global or local [8], [9] – several categories of group decision making have been described: a leadership process where one individual will propose or impose a decision that other group members will follow [10]–[15], and a voting process in which each individual indicates a direction, for instance, and after which the group will move in the direction of the majority [4], [16]–[18]. A group leader is usually defined as the individual initiating group movements but also as the individual coordinating individual during the group progression, and then mainly at the front of the progression [4], [8]–[13]. In different species of animals, leadership is not necessarily homogeneously distributed among group members [8]–[15]. Some individuals are more likely to become leaders thanks to specific internal or social traits increasing their probability of initiating a movement [10]–[12]. Studies of elephants [19], ravens [20], or fishes [21] have reported that some individuals may have a greater knowledge about their environment – which is the best site to eat or to drink – and these individuals have been observed leading the group more often than their conspecifics. In other species, individuals having a high social status, in terms of dominance or affiliation, also have a greater likelihood of being leaders. Probably the best known examples come from wolves and gorillas [10], [22] where the dominant male or couple is described as always deciding for the entire group. In Tonkean macaques, however, the most affiliated individuals – who are not necessarily the most dominant ones – seem to have a greater influence than their conspecifics in collective decision making [23].

However, one of the major factors influencing leadership should be the different physiological requirements of group members [10]–[12]. Such heterogeneity implies conflicts of interests between individuals that must be resolved in order to maintain group cohesion. Leaving the leadership to highly motivated individuals seems to be one compromise. Indeed, the moving decision seems to be taken by those with highest needs in fishes, zebras, and primates [8], [9], [14], [24]. Nevertheless, we still lack data on the way leaders emerge and the viability of the decision-making system concerning the entire group's satisfaction. Using a modelling approach, Rands et al. [12], [25] and Conradt et al. [26] showed that individuals with the highest nutrient requirements can be more prone to lead the group if there is an advantage to foraging together. Their studies were however restricted to pairs of individuals or to situations in which individuals faced two mutually exclusive target destinations only.

Here, we use a state-dependent dynamic model [27], [28] to determine how nutrient and social requirements can determine the synchronization of a group of *n* individuals, their activity budget, and the emergence of leaders. This kind of models allows us to understand how simple rules based on nutrient requirements and social factors explain synchronization between group members [27] but also segregation as shown in ungulates [28], where each individual has some requirements to satisfy (nutrient requirements such as protein, energy and water but also other social requirements and resting). We assume that if there is an advantage to being in a group, then the group members should synchronize their activities in order to stay cohesive. We assume that individuals do not know the requirements of their conspecifics (and further show that such ability may not be necessary for effective group coordination). Each individual requirement combines a reserve and a motivation that we call probability to lead. When the reserve decreases, the probability increases. At one moment, the individual with the lowest reserve – among its own needs and in comparison to other individuals – will have the highest probability to lead (these individual probabilities are compared at each time step) and will decide for the entire group on changing activity in order to fulfil its respective reserve [8], [9], [12], [25], [26]. In the next step, when the need of the previous leader is satisfied, a new leader will emerge and decide for the whole group. We applied this condition in our model in order to assess if this simple hypothesis “leading according to needs and deciding for all the group” is viable and if so, how the leadership will be distributed among group members less or more heterogeneous in their needs.

## Results

**Figure 1:**
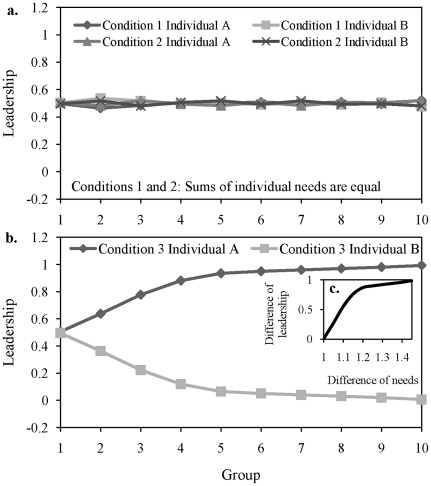
Leadership ratio according to the condition for groups of 2 individuals. Condition 1 (a.): needs are equal (*a_1_* = *a_2_* = *b_1_* = *b_2_*). Condition 2(a.): needs of each individual are different but their sums are equal between individuals (*a_1_*>*a_2_*; *b_1_*>*b_2_*; *a_1_*+*a_2_* = *b_1_*+*b_2_*). For these two conditions, the leadership is equally distributed between individuals (Kolmogorov-Smirnov test, P>0.972). Condition 3(b., c.): the sum of needs for one individual is superior to the one of individual. For Condition 3, the ratio (*a_1_*+*a_2_*)/(*b_1_*+*b_2_*) ranged from 1 (Group 1) to 1.45 (Group 10). For this condition, one individual becomes the leader when its needs are superior to the ones of its conspecifics. Fig. 1c represents the difference of leadership according to the relative difference of needs between A and B.

We first tested a group of two individuals, *A* and B, with two needs, *x_1_* and *x_2_*. We set three conditions: 1) needs are equal (*a_1_* = *a_2_* = *b_1_* = *b_2_*), 2) needs of each individual are different but their sums are equal between individuals (*a_1_*>*a_2_*; *b_1_*>*b_2_*; *a_1_*+*a_2_* = *b_1_*+*b_2_*) and 3) the sum of needs for individual *A* are always superior to the one of individual *B* (*a_1_*+*a_2_*>*b_1_*+*b_2_*). Values of needs for each tested group of each condition are detailed in table 1. We tested 10 groups for each condition. Results show that the decision system is viable, no individual dies, i.e., no individual has needs not met (reserves go to 0), whatever the tested condition. When sums of needs are equal between individuals (conditions 1 and 2), the leadership (proportion of decisions, i.e., initiations per individual) is equally distributed between the two individuals (Fig. 1A), even if, at each time step, one individual is the leader and the other one is the follower according to the reserves' difference. This result is similar to the one of the paper of Rands et al. [25] where individuals are identical. However, when the sum of needs is superior for one individual (Fig. 1B), this one becomes the leader of the pairs of individuals and the other individual becomes a follower almost all the time (Kolmogorov-Smirnov test, P<0.0001). The leadership difference between individuals increases with their relative difference of needs in a logarithmic way (curve estimation test: R^2^ = 0.96, P<0.00001; Fig. 1C). This result is similar to the one of Rands and colleagues [12]: leaders emerge when individual reserves differ.

**Table 1:** Values of daily requirements (a1, a2, b1, b2) for each individual, A and B, in each condition (1 to 3) and each group (1 to 10).

Group		1	2	3	4	5	6	7	8	9	10
Condition 1	a1	500	600	700	800	900	1100	1200	1300	1400	1500
	a2	500	600	700	800	900	1100	1200	1300	1400	1500
	b1	500	600	700	800	900	1100	1200	1300	1400	1500
	b2	500	600	700	800	900	1100	1200	1300	1400	1500
Condition 2	a1	500	600	700	800	900	1100	1200	1300	1400	1500
	a2	1500	1400	1300	1200	1100	900	800	700	600	500
	b1	1500	1400	1300	1200	1100	900	800	700	600	500
	b2	500	600	700	800	900	1100	1200	1300	1400	1500
Condition 3	a1	1000	1050	1100	1150	1200	1250	1300	1350	1400	1450
	a2	1000	1050	1100	1150	1200	1250	1300	1350	1400	1450
	b1	1000	1000	1000	1000	1000	1000	1000	1000	1000	1000
	b2	1000	1000	1000	1000	1000	1000	1000	1000	1000	1000

In a second step, we used data coming from animals in order to validate our model and to study emergence of leaders in larger groups.. According to needs of macaques, animals were divided into five categories: adult males, adult cycling females, lactating females, subadults, and juveniles. An individual has five requirements to satisfy: water, protein, energy, resting, and socializing [29]–[34]. The nutrient requirements of an individual (water, protein and energy) depend on its body mass whilst social and resting needs did not [31]–[34]. We chose to include social activity in the model because many social species spend time maintaining their relationships and group cohesion [31]–[35]. Group composition (table 2) and individual characteristics (table 3) are based on data on macaques and are detailed in the method section. We tested 10 groups of 5, 10 and 20 individuals with same needs (individuals of the same category and with the same body mass) and 10 ones with different needs (individuals of both different categories and different body masses).

**Table 2:** Mean, minimum, and maximum number of individuals per category for *n* = 10 individuals per group.

	Mean±SD	Minimum	Maximum
Adult male	1.6±0.7	1	3
Adult cycling female	3.1±1.3	2	6
Lactating female	1.7±1.3	0	4
Subadults	1.9±1.3	0	4
Juveniles	1.8±0.9	0	3

**Table 3:** Mean individual weight and requirements for each category and each need.

		Requirement				
	weight (kg)	protein (g.day^−1^)	energy (KJ.day-1)	water (ml.day^−1^)	social time (minutes.day^−1^)	resting time (minutes.day^−1^)
Adult males	15.18±1.34	38.55±3.40	5313±889	1269.08±212.34	100±50.41	87.50±55.71
Females	9.62±2.39	24.44±6.07	3191±792	762.24±189.20	112.73±19.89	50.91±33.52
Lactating females	9.85±1.04	30.06±2.63	6059±1191	1447.23±284.50	86.67±30.688	106.67±37.41
Subadults	4.43±0.66	11.27±1.67	1471±219	351.40±52.35	170.53±30.09	30±28.72
Juveniles	3.33±1.43	8.46±3.63	1105±474	264.00±113.32	176.67±14.14	80.83±33.80

**Figure 2:**
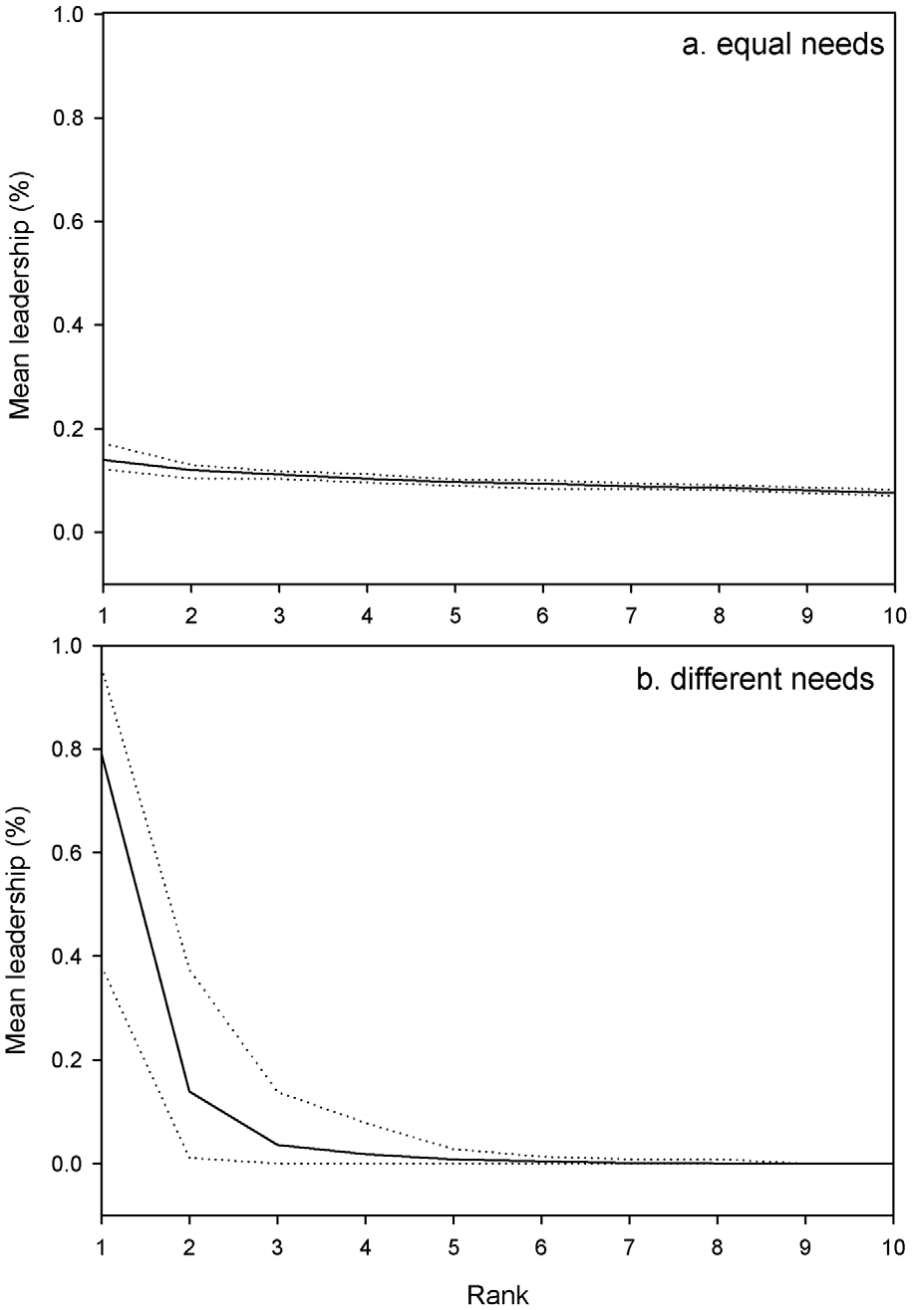
Mean leadership proportion or ratio (solid line) according to individuals with (a) equal needs and (b) different needs in groups of 10 individuals. Individuals are ranked from the individual with the highest leadership proportion to the one with the lowest leadership proportion. The upper and lower dotted lines indicate respectively the maximum and minimum leadership proportions for each rank. The leadership proportion is different between individuals whatever the condition.

**Figure 3:**
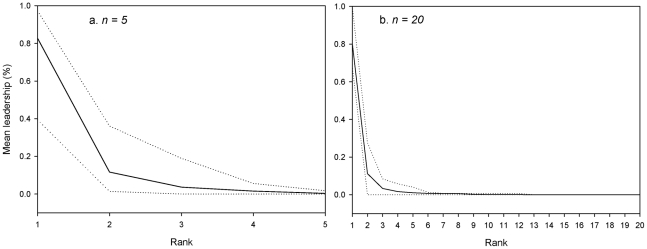
Mean leadership proportion or ratio (solid line) according to individual in groups of (a) 5 and (b) 20 individuals having different needs. Individuals are ranking from the individual with the higher leadership proportion to the one with the lower leadership proportion. The up and down dotted lines indicate respectively the maximum and minimum leadership proportions for each rank.

Simulations showed that the system – leadership by those in need – is sustainable in groups of 5, 10 and 20 individuals. All individual requirements are satisfied at the end of simulations, whatever the group composition. Moreover, the group activity budget is fairly similar to the activity budget of wild primate groups (27.3±1.7% of time devoted to moving, 33.8±1.7% to foraging, 21.7±0.7% to resting, and 17.2±3.1% to socializing). All individuals could become leaders but the distribution of the leadership proportion is not the same according to the requirements' heterogeneity (equal or different needs; Kruskal-Wallis test, P<0.001). In groups with similar needs, the proportion of leadership differs weakly between individuals and is about 10% per individual. The relation between leadership proportion and rank (i.e., individuals were ranked from the most frequent leader to the less frequent one) is linear (linear curve estimation test: R^2^ = 0.92, F_1,8_ = 93.05, P<0.00001, y = −0.0006x+0.1339). The leadership is about 14% for the individual that decides the most and 7.6% for the individual that decide the least (Fig. 2A). This result corresponds to the equiprobability of being leader per individual (proportion divided by the number of individuals per group). On the other hand, the leadership is not equally distributed in heterogeneous groups. The relation between the proportion of leadership and individuals is exponential (exponential curve estimation test: R^2^ = 0.83, F_1,8_ = 38.07, P = 0.0002, y = 3.5727e^−4.602x^, Fig. 2B), with one individual being responsible for 38% to 95% of decisions per group, while some individuals decide only in 0.0003% to 0.0007% of cases per group. We obtained the same relationship with groups of 5 (exponential curve estimation test; R^2^ = 0.97, F_1,3_ = 12.81, P<0.00001, y = 0.9825x^−3.207^, Fig. 3A) and 20 individuals (exponential curve estimation test; R^2^ = 0.96, F_1,18_ = 498.95, P<0.00001, y = 11.48x^−4.86^, Fig. 3B).

**Figure 4:**
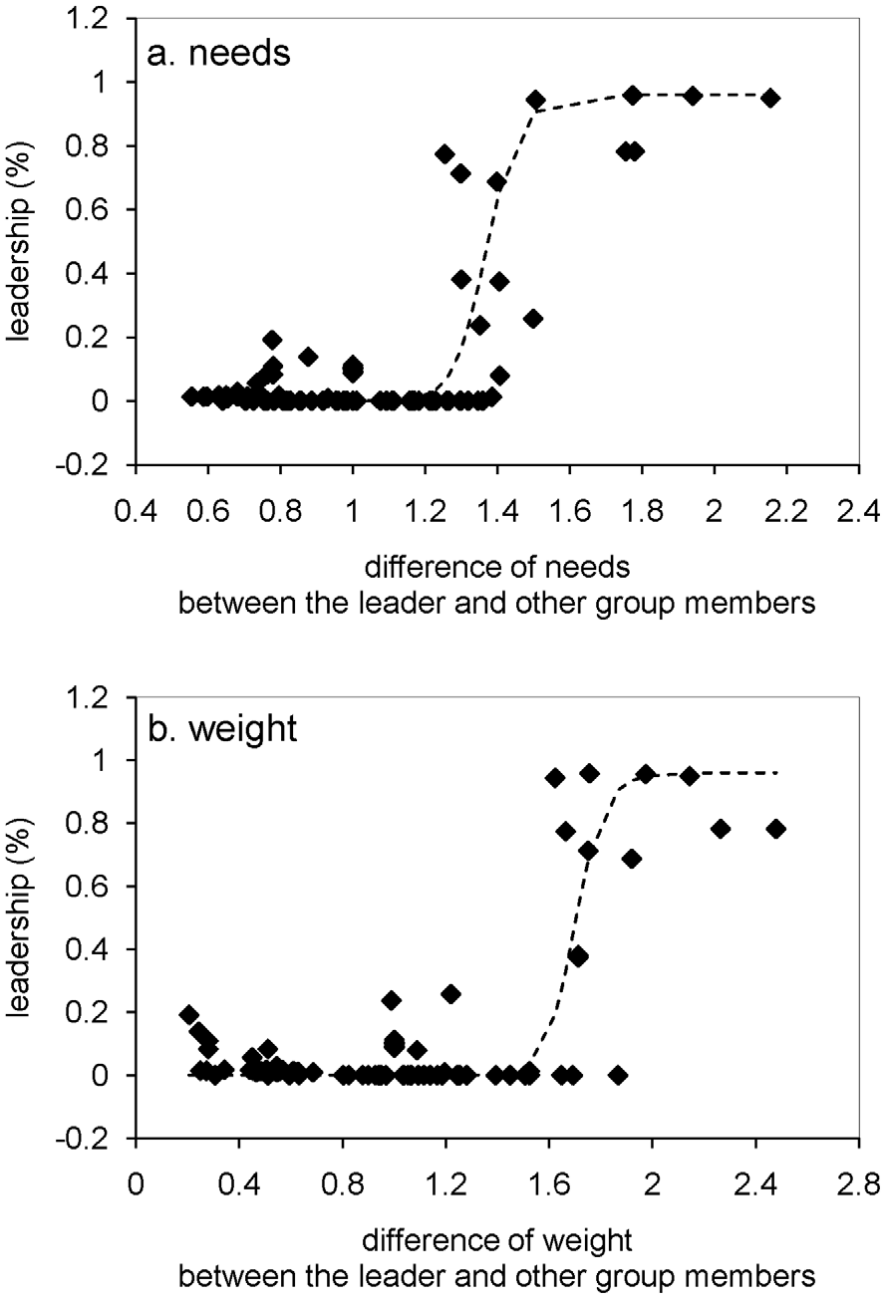
Relation between the leadership proportion and (a) the difference in needs and (b) the difference in mass for each individual and the other group members in groups of 10 individuals. These functions are both sigmoid. N = 110. Each point represents characteristics of one individual in each group (ten groups with different needs and one with equal needs are represented).

**Figure 5:**
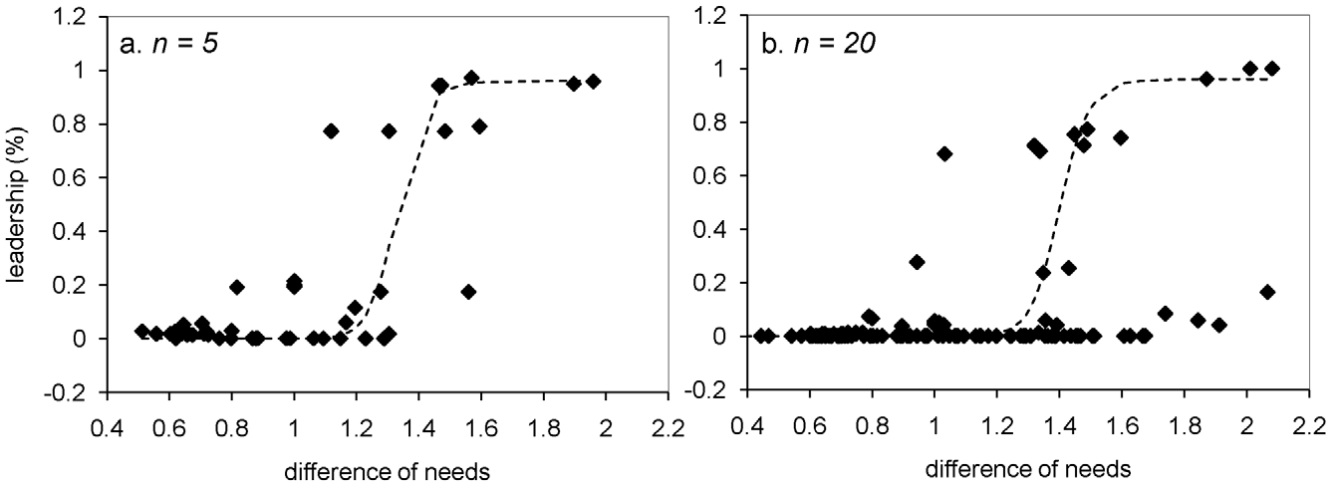
Relation between the leadership proportion and the differences in need in groups of (a) 5 and (b) 20 individuals. These functions are both sigmoid. Each point represents characteristics of one individual in each group (ten groups with different needs and one with equal needs are represented).

We compared this unequally distributed leadership to the requirements of individuals in order to understand how so many differences can emerge in heterogeneous groups. We calculated the relative difference in requirements (corresponding to the highest probability to lead) between each leader and other individuals. The relationship between the leadership and this difference in requirements follows a sigmoid curve (
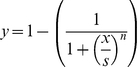
 with a threshold *S* of 1.37 and a minimal *n* value of 30; curve estimation test: R^2^ = 0.71, F_1,108_ = 269.72, P<0.00001; Fig. 4A). The *n* value represents the sensitivity of the process. The higher the *n* value is, the more sensitive the process is (quick and sudden transition between the two states). In our context, this means that one individual becomes the most frequent and prominent leader of a group as soon as one of its requirements exceed about 137% of those of one of its conspecifics. This transition between equally distributed leadership and one exclusive leader is highly non-linear, given the n value we observed. The same sigmoid law is observed between the proportion of leadership and the body mass of individuals (sigmoid curve estimation test: R^2^ = 0.66, F_1,108_ = 205.73, P<0.00001; Fig. 4B). When the mass of an individual is more than 170% (S = 1.7, n = 30) of those of its conspecifics, this individual is the main group leader. Except for lactating females, requirements and then leadership are related to body mass in about 60% of cases. The rest of the decisions concern resting and socializing and are not related to mass. We obtain similar results for groups of 5 (sigmoid curve estimation test: R^2^ = 0.71, F_1,53_ = 111.52, P<0.00001, 
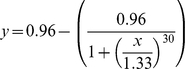
, Fig. 5A) and 20 individuals (sigmoid curve estimation test: R^2^ = 0.16, F_1,218_ = 42.13, P<0.00001, 
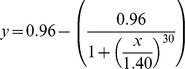
, Fig. 5B). For 8 out of 10 groups of 20 individuals, 4.6±2.2 group members were never leader. They were satisfied by following their conspecifics.

## Discussion

Leading by those highest in need resembles the results obtained by Rands et al. [25], where the individual with the lower reserve spontaneously becomes the leader. Moreover, a recent study by Conradt et al. [26] showed that a small minority of individuals with strong needs are more prone to lead the group than a larger majority of individuals with few needs. However, it is the first time that a threshold [2], [18], [36] has been demonstrated concerning the emergence of leadership. The decision-making system implies high differences in leadership proportion whilst relatively small differences are observed in the requirements of individuals. The threshold we obtained in this study is probably dependent on 1) the group structure of primates (one or a small number of males compared to the other categories) [35] and 2) to the physiology of primates [31]–[34]. Indeed, in primates, and especially in macaques, a sexual dimorphism exists and males may reach a mass 150 to 200% superior to the one of females.

**Figure 6:**
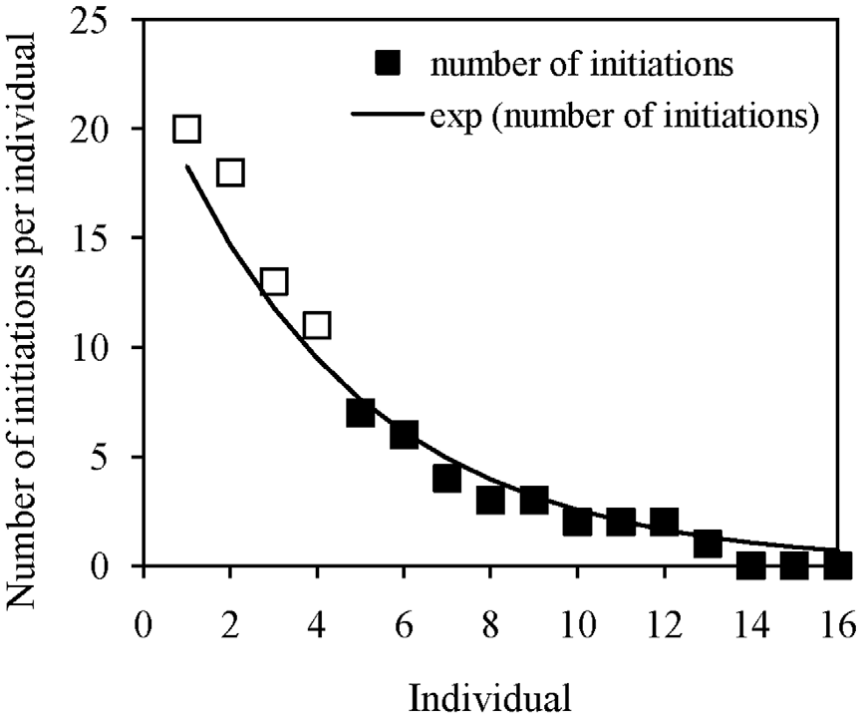
Number of initiations per individual in a group of baboons studied by Stueckle and Zinner [**36**]. Black squares represent females, white squares represent males. The distribution of initiations follows an exponential curve as (black line) determined by the study model (curve estimation test: R^2^ = 0.99, P<0.00001, y = 22.73e^−0.219x^).

Several authors suggested that dominant individuals are the only leaders in several species [9]–[12], [14], [22], [23]. The dominance is however strongly correlated to the body mass and then to the nutrient requirements of animals [10]. This indirect effect of dominance on leadership, through the needs and then the probability to initiate a movement, needs to be taken into account in subsequent studies testing dominance effects. For instance, two field studies in baboons showed that the main leader – the individual initiating most of movements – was the dominant male. However this male is also certainly the biggest individual in the group. In the study of Stueckle and Zinner [36], the four males of the group, bigger than females, are the ones initiating the most of movements (Fig. 6). Moreover, the distribution of leadership also follows an exponential as the one in the study model. We may suggest that the slope of this exponential distribution of leadership will be less or more important according to the group composition. This slope would be around 0 when the group is homogeneous and increases with group heterogeneity.

The non-linear differences in leadership among group members eventually emerge from two simple rules: individuals need to remain cohesive and the individual with the lowest reserve at one moment decides for the group [2], [3], [24]–[26]. Mechanisms of coordination and cohesion do not need complex signalling or complex cognitive ability [2], [3], [13], [24]. The emergence of a unique leader may also occur when decisions are not necessarily imposed on other group members but because other individuals do not express the necessity to move or to make a decision. An individual becomes a leader because its conspecifics decide to follow it [8], [9]. This outcome may make important contributions to our understanding of decision making in animal and human societies.

## Materials and Methods

### Models'parameters

The model was developed in Netlogo 3.15 [37]. The model and model's procedures can be found in the supplementary material “Dataset S1”. One time-step in the simulation represents one minute. We defined the probability to lead α for the requirement A and the individual *i* as:
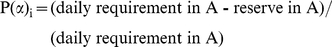
The probability to lead for the individual *i* is:
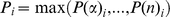
In this way, the probability to lead can vary between 1 (highest probability to lead, weakest reserve) and 0 (weakest probability to lead, highest reserve). Each reserve is bounded by a maximum above which each group member cannot gain further reserves and a minimum at which each group member is assumed to die if it is reached. At each timestep (equal to one minute), each reserve of each individual decreases (i.e., expenditure) depending on the individual category and the current activity. This reserve decrease will increase the individual probability to lead. In order to fulfil this reserve, the individual should have to carry out the corresponding activity (i.e., intake). This gain may be done by becoming a leader or by following the leader. We implement optimal foraging decisions in the model: when an individual decides to forage, it will forage until its reserve has been fulfilled. After the end of each activity period, the individual with the highest probability to lead *P_i_* becomes the new leader. Individuals have a walking speed of 0.4m.s^−1^.

The group environment is two-dimensional environment of 96×96 connected cells. Each cell represent one meter. Each cell has four immediate neighbours and the sides of the arena were joined to form a torus. The number of areas where animals fulfil their reserves is two for the first model with two individuals having two needs and four for the model from 5 to 20 individuals having five needs (see details below). At the start of a simulation, individuals are at the same distance of each area (i.e., at the middle of the torus). According to the distribution of areas inside the torus, groups have a travel distance between two areas ranging from a minimum of 25 meters to a maximum of 75 meters. This range fits with travel distances in primate species of similar body mass and similar group size [4], [38]–[41]. Positions of areas were fixed in our model but this does not affect results since variability among needs – what is the highest need and the weakest one – is much more important between individuals and groups. This means that the areas corresponding for instance to the two highest needs for an individual are not always the closest ones. There is no intragroup competition in this model: all individuals can occupy the same area. We run 1000 simulations for each group. A simulation stops when one reserve of one individual reaches 0 or after 90 days.

### Model with two individuals having two needs

The two individuals have two needs and thus two daily requirements. Values of these requirements for each condition and each individual are described in table 1. We tested ten different groups for each condition. Expenditures of each reserve are 0.07±0.035 units.min^−1^. Intakes are 10 units.min^−1^. The environment is composed of two areas, one for each requirement. Individuals have to move to the respective area to fulfil each reserve.

### Model with five to 20 individuals with five needs

According to data in macaques, the daily protein requirement is estimated to 2.54g.day^−1^.kg^−1^, daily energy requirement to 351.7Kcal.day^−1^.kg^−1^, and daily water requirement to 0.24ml.KJ^−1^, except for lactating females for which these requirements are higher than the ones of non lactating females (about 125% for proteins and 200% for energy and water of requirements of non lactating females) [31]–[34]. Social and resting times are not dependent on body mass. Individual expenditure per need and activity is described in table 4. Details about individual intake rate per need are in table 5.

**Table 4:** Individual expenditure per activity for each need.

	Expenditure				
	protein (g.min^−1^.kg^−1^)	energy (KJ.min^−1^.kg^−1^)	water (ml.min^−1^.kg^−1^)	social time (min.min^−1^)	resting time (min.min^−1^)
Foraging	0.0027±0.0013	0.29±0.15	0.07±0.035	social time requirement/720	resting time requirement/720
Walking		0.24±0.10	0.0575±0.023		
Socializing		0.29±0.15	0.07±0.035		
Resting		0.10±0.05	0.025±0.012		

**Table 5:** Mean individual intake rate categories for each need.

	Intake				
	protein (g.min^−1^)	energy (KJ.min^−1^)	water (ml.min^−1^)	social time (min.min^−1^)	resting time (min.min^−1^)
Males	0.217±0.108	41.9±23.1	50±25	1.0±0.5	1.0±0.5
Females	0.217±0.108	38.9±18.7			
Lactating females	0.217±0.108	38.2±20.2			
Subadults	0.202±0.101	28.4±15.2			
Juveniles	0.126±0.63	23.7±11.2			

The environment is composed of four areas: one area for foraging for proteins, one area for foraging for energy, one waterhole, and one resting site [42]. When individuals need energy, proteins, or water, they have to move toward the respective areas. Until the group is in a specific activity among the five ones (eating proteins, eating energy, drinking water, resting or socializing), each individual gains a certain amount of the requirement according to its category (table 5). Concerning resting, individuals need to go to the resting site for the night (at the 720^th^ time-step and for 720 time-steps), but during the day they can rest in any area. The same rule applies to socializing. Concerning resting and socializing activity, we fixed a minimal period of 5 minutes for doing these activities.

### Statistics

Differences in leadership between individuals were tested using a Kolmogorov-Smirnov test for groups of 2 individuals and a Kruskall-Wallis test for groups from 5 to 20 individuals. The relations between the proportion of leadership and differences in needs or mass were determined through a curve estimation test. We compared observed curves to exponential, linear and sigmoid ones. Only theoretical curves best fitting with observed data are indicate in results. Analyses were performed in SPSS 10.00. α was set at 0.05. Means were ± S.E.M.

## Supporting Information

Dataset S1 This file contains the model used for this publication and its related files. The file “modele beta min.nlogo” is the model used for this publication. Algorithms can be seen in the “procedures” window. “attributes.txt” file is used to implement individual characteristics in the model. Row 1 corresponds to the identity of agents. Row 2 represents the body mass. Row 3 is the daily protein requirement. Row 4 is the daily energy requirement. Row 5 is the protein intake rate. Row 6 is the energy intake rate. Row 7 is the daily water requirement. Row 8 is the daily social time requirement. Row 9 is the daily resting time requirement. Row 10 is the category of individuals (male, female, etc.). “Links.txt” file is used to implement social relationships of individuals in the model. This variable is not used and analyzed in this study. “activitybudget.doc” file is used to score group activity budget per day. “highestvalue.doc file” is used to score which individual has the highest motivation at the end of the day and what is this motivation. “idleaderfrequency.doc” is used to score the frequency of leadership per individual during all the simulation.
(0.35 MB ZIP)
