# Supplementary material for: Differences in Nutrient Requirements Imply a Non-Linear Emergence of Leaders in Animal Groups
Source: PLoS Comput Biol. 2010 Sep 2;6(9):e1000917. doi: 10.1371/journal.pcbi.1000917 (PMC2932680; doi:10.1371/journal.pcbi.1000917)
Supplement: Dataset S1 — This file contains the model used for this publication and its related files. The file “modele beta min.nlogo” is the model used for this publication. Algorithms can be seen in the “procedures” window. “attributes.txt” file is used to implement individual characteristics in the model. Row 1 corresponds to the identity of agents. Row 2 represents the body mass. Row 3 is the daily protein requirement. Row 4 is the daily energy requirement. Row 5 is the protein intake rate. Row 6 is the energy intake rate. Row 7 is the daily water requirement. Row 8 is the daily social time requirement. Row 9 is the daily resting time requirement. Row 10 is the category of individuals (male, female, etc.). “Links.txt” file is used to implement social relationships of individuals in the model. This variable is not used and analyzed in this study. “activitybudget.doc” file is used to score group activity budget per day. “highestvalue.doc file” is used to score which individual has the highest motivation at the end of the day and what is this motivation. “idleaderfrequency.doc” is used to score the frequency of leadership per individual during all the simulation. (0.35 MB ZIP) [file pcbi.1000917.s001.zip › Read it first.doc]

The file “modele beta min.nlogo” is the model used for this publication. Algorithms can be seen in the “procedures” window.

“attributes.txt” file is used to implement individual characteristics in the model. Row 1 corresponds to the identity of agents. Row 2 represents the body mass. Row 3 is the daily protein requirement. Row 4 is the daily energy requirement. Row 5 is the protein intake rate. Row 6 is the energy intake rate. Row 7 is the daily water requirement. Row 8 is the daily social time requirement. Row 9 is the daily resting time requirement. Row 10 is the category of individuals (male, female, etc.).

“Links.txt” file is used to implement social relationships of individuals in the model. This variable is not used and analyzed in this study.

“activitybudget.doc” file is used to score group activity budget per day.

“highestvalue.doc file” is used to score which individual has the highest motivation at the end of the day and what is this motivation.

“”idleaderfrequency.doc” is used to score the frequency of leadership per individual during all the simulation.

**For those not having Netlogo, please find the model’s procedures below:**

breed [nodes node]

nodes-own [node-id activity dailyproteinrate dailyproteinrequirement socialbout weight needprotein dailyenergyrate dailyenergyrequirement needenergy needtodrink waterintakeneeded dailydrinkrate socialtime socialtimeneeded needtosocial restingtime restingtimeneeded needtorest proteinintakerate energyintakerate category highestvalue] ;; activity 0: simulation start; activity 1 : at sleeping site (rest); activity 2: at protein site: (forage protein); activity 3: at water zone(drink); activity 4: at energysite (forage energy)

breed [links link]

links-own [strength]

patches-own [zone]

globals [simulations leader links-list clock adhesion-time mimetic-coefficient day dailypathlength socialbudget restingbudget foragingbudget movingbudget dead]

to setup

ca

set simulations 1

ask patch -1 1 [ set zone 1

set pcolor brown];; sleeping zone

ask patch 1 1 [ set zone 2

set pcolor green];; protein zone

ask patch 1 -1 [ set zone 3

set pcolor blue];; water zone

ask patch -1 -1 [ set zone 4

set pcolor grey];; energy zone

end

to simulate

ct

import-network

set clock 1

set day 0

set socialbudget 0

set restingbudget 0

set foragingbudget 0

set movingbudget 0

set adhesion-time 1

ask nodes [

set color red

set activity 0

set dailyproteinrate (dailyproteinrequirement - (random-normal 0.0008 0.0004 * weight * 12 * 60))

set dailyenergyrate (dailyenergyrequirement - (0.05 * weight * 12 * 60))

set dailydrinkrate (waterintakeneeded - (0.05 * weight * 12 * 60))

set socialtime socialtimeneeded

set restingtime restingtimeneeded

set dailypathlength 0

set socialbout 0

]

output-write "simulation " + simulations

output-print " "

dailyactivity

output-print "number of days"

output-print day

output-print "category of individual with highestvalue"

output-print category-of max-one-of nodes [highestvalue]

output-print "values of highestvalue"

output-print value-from (max-one-of nodes [highestvalue]) [highestvalue]

file-open "highestvalue.doc"

file-type "sim" + simulations + " : "

file-write day

file-write value-from (max-one-of nodes [highestvalue]) [highestvalue]

file-write category-of max-one-of nodes [highestvalue]

file-write node-id-of max-one-of nodes [highestvalue]

if value-from (max-one-of nodes [highestvalue]) [highestvalue] = value-from (max-one-of nodes [highestvalue]) [needprotein] [file-write "protein"]

if value-from (max-one-of nodes [highestvalue]) [highestvalue] = value-from (max-one-of nodes [highestvalue]) [needenergy] [file-write "energy"]

if value-from (max-one-of nodes [highestvalue]) [highestvalue] = value-from (max-one-of nodes [highestvalue]) [needtodrink] [file-write "water"]

if value-from (max-one-of nodes [highestvalue]) [highestvalue] = value-from (max-one-of nodes [highestvalue]) [needtorest] [file-write "rest"]

if value-from (max-one-of nodes [highestvalue]) [highestvalue] = value-from (max-one-of nodes [highestvalue]) [needtosocial] [file-write "social"]

file-print " "

file-close

ifelse simulations < simulations-number

[set simulations simulations + 1

simulate]

[stop simulate]

end

to dailyactivity

ask nodes

[set needprotein ((dailyproteinrequirement - dailyproteinrate)/ dailyproteinrequirement)

set needenergy ((dailyenergyrequirement - dailyenergyrate)/ dailyenergyrequirement)

set needtodrink ((waterintakeneeded - dailydrinkrate)/ waterintakeneeded)

set needtosocial ((socialtimeneeded - socialtime)/ socialtimeneeded)

set needtorest ((restingtimeneeded - restingtime)/ restingtimeneeded)

if (needprotein >= needenergy) and (needprotein > needtodrink) and (needprotein >= needtosocial) and (needprotein >= needtorest)

[set highestvalue needprotein]

if (needenergy > needprotein) and (needenergy > needtodrink) and (needenergy >= needtosocial) and (needenergy >= needtorest)

[set highestvalue needenergy]

if (needtodrink >= needprotein) and (needtodrink >= needenergy) and (needtodrink >= needtosocial) and (needtodrink >= needtorest)

[set highestvalue needtodrink]

if (needtosocial > needprotein) and (needtosocial > needenergy) and (needtosocial > needtodrink) and (needtosocial > needtorest)

[set highestvalue needtosocial]

if (needtorest > needprotein) and (needtorest > needenergy) and (needtorest > needtodrink) and (needtorest >= needtosocial)

[set highestvalue needtorest]]

if (needprotein-of one-of nodes >= 1) or (needenergy-of one-of nodes >= 1) or (needtodrink-of one-of nodes >= 1) or (needtosocial-of one-of nodes >= 1) or (needtorest-of one-of nodes >= 1)

[ stop dailyactivity]

if day = 90 [stop dailyactivity]

set leader max-one-of nodes [highestvalue]

file-open "idleaderfrequency.doc"

file-write node-id-of leader

file-write category-of leader

file-print " "

file-close

ask leader

[if highestvalue = needprotein

[forageprotein]

if highestvalue = needenergy

[forageenergy]

if highestvalue = needtodrink

[drink]

if highestvalue = needtosocial

[social]

if highestvalue = needtorest

[rest]

]

ifelse clock > 720

[night]

[dailyactivity]

end

to forageprotein

ask nodes

[set needprotein ((dailyproteinrequirement - dailyproteinrate)/ dailyproteinrequirement)

set needenergy ((dailyenergyrequirement - dailyenergyrate)/ dailyenergyrequirement)

set needtodrink ((waterintakeneeded - dailydrinkrate)/ waterintakeneeded)

set needtosocial ((socialtimeneeded - socialtime)/ socialtimeneeded)

set needtorest ((restingtimeneeded - restingtime)/ restingtimeneeded)

]

ask leader

[ifelse patch-here = patch 1 1

[fd 0

set dailyproteinrate (dailyproteinrate + proteinintakerate - (random-normal 0.0027 0.0013 * weight))

set dailyenergyrate (dailyenergyrate - (random-normal 0.0696 0.0348 * weight))

set restingtime (restingtime - (restingtimeneeded / 720))

set dailydrinkrate (dailydrinkrate - (random-normal 0.059 0.029 * weight))

set socialtime (socialtime - (socialtimeneeded / 720))

set activity 2

set foragingbudget foragingbudget + 1

set clock clock + 1

ask nodes with [node-id != node-id-of leader]

[fd 0

set dailyproteinrate (dailyproteinrate + proteinintakerate - (random-normal 0.0027 0.0013 * weight))

set dailyenergyrate (dailyenergyrate - (random-normal 0.0696 0.0348 * weight))

set dailydrinkrate (dailydrinkrate - (random-normal 0.059 0.029 * weight))

set restingtime (restingtime - (restingtimeneeded / 720))

set socialtime (socialtime - (socialtimeneeded / 720))]

if dailyproteinrate < dailyproteinrequirement

[forageprotein]

]

[facexy 1 1

fd 1

set activity 2

set clock clock + 1

set movingbudget movingbudget + 1

set dailyproteinrate (dailyproteinrate - random-normal 0.0027 0.0013 * weight)

set dailydrinkrate (dailydrinkrate - (random-normal 0.059 0.029 * weight))

set dailyenergyrate (dailyenergyrate - (random-normal 0.0696 0.0348 * weight))

set dailypathlength (dailypathlength + 25)

set restingtime (restingtime - (restingtimeneeded / 720))

set socialtime (socialtime - (socialtimeneeded / 720))

ask nodes with [node-id != node-id-of leader]

[	facexy 1 1

fd 1

set activity 2

set dailyproteinrate (dailyproteinrate - (random-normal 0.0027 0.0013 * weight))

set dailydrinkrate (dailydrinkrate - (random-normal 0.059 0.029 * weight))

set dailyenergyrate (dailyenergyrate - (random-normal 0.0696 0.0348 * weight))

set restingtime (restingtime - (restingtimeneeded / 720))

set socialtime (socialtime - (socialtimeneeded / 720))

]

]

]

end

to forageenergy

ask nodes

[set needprotein ((dailyproteinrequirement - dailyproteinrate)/ dailyproteinrequirement)

set needenergy ((dailyenergyrequirement - dailyenergyrate)/ dailyenergyrequirement)

set needtodrink ((waterintakeneeded - dailydrinkrate)/ waterintakeneeded)

set needtosocial ((socialtimeneeded - socialtime)/ socialtimeneeded)

set needtorest ((restingtimeneeded - restingtime)/ restingtimeneeded)

]

ask leader

[ifelse patch-here = patch -1 -1

[fd 0

set dailyenergyrate (dailyenergyrate + energyintakerate - (random-normal 0.0696 0.0348 * weight))

set dailyproteinrate (dailyproteinrate - (random-normal 0.0027 0.0013 * weight))

set dailydrinkrate (dailydrinkrate - (random-normal 0.059 0.029 * weight))

set restingtime (restingtime - (restingtimeneeded / 720))

set socialtime (socialtime - (socialtimeneeded / 720))

set activity 4

set foragingbudget foragingbudget + 1

set clock clock + 1

ask nodes with [node-id != node-id-of leader]

[fd 0

set dailyenergyrate (dailyenergyrate + energyintakerate - (random-normal 0.0696 0.0348 * weight))

set dailyproteinrate (dailyproteinrate - (random-normal 0.0027 0.0013 * weight))

set restingtime (restingtime - (restingtimeneeded / 720))

set dailydrinkrate (dailydrinkrate - (random-normal 0.059 0.029 * weight))

set socialtime (socialtime - (socialtimeneeded / 720))

set activity 4]

if dailyenergyrate < dailyenergyrequirement

[forageenergy]

]

[facexy -1 -1

fd 1

set activity 4

set clock clock + 1

set movingbudget movingbudget + 1

set dailyproteinrate (dailyproteinrate - (random-normal 0.0027 0.0013 * weight))

set dailydrinkrate (dailydrinkrate - (random-normal 0.059 0.029 * weight))

set dailyenergyrate (dailyenergyrate - (random-normal 0.0696 0.0348 * weight))

set dailypathlength (dailypathlength + 25)

set restingtime (restingtime - (restingtimeneeded / 720))

set socialtime (socialtime - (socialtimeneeded / 720))

ask nodes with [node-id != node-id-of leader]

[facexy -1 -1

fd 1

set activity 4

set dailyproteinrate (dailyproteinrate - (random-normal 0.0027 0.0013 * weight))

set dailyenergyrate (dailyenergyrate - (random-normal 0.0696 0.0348 * weight))

set dailydrinkrate (dailydrinkrate - (random-normal 0.059 0.029 * weight))

set restingtime (restingtime - (restingtimeneeded / 1440))

set socialtime (socialtime - (socialtimeneeded / 720))

]

]

]

end

to rest

ask nodes

[set needprotein ((dailyproteinrequirement - dailyproteinrate)/ dailyproteinrequirement)

set needenergy ((dailyenergyrequirement - dailyenergyrate)/ dailyenergyrequirement)

set needtodrink ((waterintakeneeded - dailydrinkrate)/ waterintakeneeded)

set needtosocial ((socialtimeneeded - socialtime)/ socialtimeneeded)

set needtorest ((restingtimeneeded - restingtime)/ restingtimeneeded)

]

ask leader

[fd 0

set activity 1

set clock clock + 1

set restingbudget restingbudget + 1

set restingtime restingtime + 1

set socialtime (socialtime - (socialtimeneeded / 720))

set dailydrinkrate (dailydrinkrate - (random-normal 0.059 0.029 * weight))

set dailyenergyrate (dailyenergyrate - (random-normal 0.025 0.012 * weight))

set dailyproteinrate (dailyproteinrate - (random-normal 0.0008 0.0004 * weight))

ask nodes with [node-id != node-id-of leader]

[ fd 0

set dailyenergyrate (dailyenergyrate - (random-normal 0.025 0.012 * weight))

set dailyproteinrate (dailyproteinrate - (random-normal 0.0008 0.0004 * weight))

set dailydrinkrate (dailydrinkrate - (random-normal 0.059 0.029 * weight))

set activity 1

set restingtime restingtime + 1

set socialtime (socialtime - (socialtimeneeded / 720))]

]

end

to social

ask nodes

[set needprotein ((dailyproteinrequirement - dailyproteinrate)/ dailyproteinrequirement)

set needenergy ((dailyenergyrequirement - dailyenergyrate)/ dailyenergyrequirement)

set needtodrink ((waterintakeneeded - dailydrinkrate)/ waterintakeneeded)

set needtosocial ((socialtimeneeded - socialtime)/ socialtimeneeded)

set needtorest ((restingtimeneeded - restingtime)/ restingtimeneeded)

]

ask leader

[fd 0

set activity 5

set socialbout socialbout + 1

set clock clock + 1

set socialbudget socialbudget + 1

set dailyproteinrate (dailyproteinrate - (random-normal 0.0027 0.0013 * weight))

set dailydrinkrate (dailydrinkrate - (random-normal 0.059 0.029 * weight))

set dailyenergyrate (dailyenergyrate - (random-normal 0.07 0.035 * weight))

set restingtime (restingtime - (restingtimeneeded / 720))

set socialtime (socialtime + 1)

ask nodes with [node-id != node-id-of leader]

[fd 0

set activity 5

set dailyproteinrate (dailyproteinrate - (random-normal 0.0027 0.0013 * weight))

set dailydrinkrate (dailydrinkrate - (random-normal 0.059 0.029 * weight))

set dailyenergyrate (dailyenergyrate - (random-normal 0.07 0.035 * weight))

set restingtime (restingtime - (restingtimeneeded / 720))

set socialtime (socialtime + 1)]

if socialbout < 5 [social]

set socialbout 0]

end

to drink

ask nodes

[set needprotein ((dailyproteinrequirement - dailyproteinrate)/ dailyproteinrequirement)

set needenergy ((dailyenergyrequirement - dailyenergyrate)/ dailyenergyrequirement)

set needtodrink ((waterintakeneeded - dailydrinkrate)/ waterintakeneeded)

set needtosocial ((socialtimeneeded - socialtime)/ socialtimeneeded)

set needtorest ((restingtimeneeded - restingtime)/ restingtimeneeded)

]

ask leader

[ifelse patch-here = patch 1 -1

[fd 0

set activity 3

set clock clock + 1

set foragingbudget foragingbudget + 1

set dailydrinkrate (dailydrinkrate + random-normal 50 25)

set restingtime (restingtime - (restingtimeneeded / 720))

set socialtime (socialtime - (socialtimeneeded / 720))

set dailyenergyrate (dailyenergyrate - (random-normal 0.0696 0.0348 * weight))

set dailyproteinrate (dailyproteinrate - (random-normal 0.0027 0.0013 * weight))

ask nodes with [node-id != node-id-of leader]

[ fd 0

set dailyenergyrate (dailyenergyrate - (random-normal 0.0696 0.0348 * weight))

set dailydrinkrate (dailydrinkrate + random-normal 50 25)

set dailyproteinrate (dailyproteinrate - (random-normal 0.0027 0.0013 * weight))

set restingtime (restingtime - (restingtimeneeded / 720))

set socialtime (socialtime - (socialtimeneeded / 720))

set activity 3

set dailydrinkrate (dailydrinkrate + 1)]

if dailydrinkrate < waterintakeneeded [drink]

]

[facexy 1 -1

fd 1

set activity 3

set clock clock + 1

set movingbudget movingbudget + 1

set dailyproteinrate (dailyproteinrate - (random-normal 0.0027 0.0013 * weight))

set dailyenergyrate (dailyenergyrate - (random-normal 0.0696 0.0348 * weight))

set dailypathlength (dailypathlength + 25)

set restingtime (restingtime - (restingtimeneeded / 720))

set socialtime (socialtime - (socialtimeneeded / 720))

ask nodes with [node-id != node-id-of leader]

[	facexy 1 -1

fd 1

set activity 3

set dailyproteinrate (dailyproteinrate - (random-normal 0.0027 0.0013 * weight))

set dailyenergyrate (dailyenergyrate - (random-normal 0.07 0.035 * weight))

set restingtime (restingtime - (restingtimeneeded / 720))

set socialtime (socialtime - (socialtimeneeded / 720))

]

]]

end

to night

ask nodes

[set needprotein ((dailyproteinrequirement - dailyproteinrate)/ dailyproteinrequirement)

set needenergy ((dailyenergyrequirement - dailyenergyrate)/ dailyenergyrequirement)

set needtodrink ((waterintakeneeded - dailydrinkrate)/ waterintakeneeded)

set needtosocial ((socialtimeneeded - socialtime)/ socialtimeneeded)

set needtorest ((restingtimeneeded - restingtime)/ restingtimeneeded)

]

ask nodes

[ifelse patch-here = patch -1 1

[fd 0

set activity 1

set restingtime restingtimeneeded

set dailyenergyrate (dailyenergyrate - (random-normal 0.0012 0.0006 * weight * 720))

set dailyproteinrate (dailyproteinrate - (random-normal 0.05 0.025 * weight * 720))

]

[facexy -1 1

fd 1

set activity 11

set dailyproteinrate (dailyproteinrate - (random-normal 0.07 0.035 * weight))

set dailyenergyrate (dailyenergyrate - (random-normal 0.0027 0.0013 * weight))

set dailypathlength (dailypathlength + 25)

set restingtime (restingtime - (restingtimeneeded / 720))

set socialtime (socialtime - 1)

]]

output-print "day" + day

output-print "movingbudget :"

output-print movingbudget

output-print "restingbudget :"

output-print restingbudget

output-print "foragingbudget :"

output-print foragingbudget

output-print "socialbudget :"

output-print socialbudget

file-open "activitybudget.doc"

file-write movingbudget

file-write foragingbudget

file-write socialbudget

file-write restingbudget

file-write dailypathlength

file-print " "

file-close

set clock 0

set movingbudget 0

set restingbudget 0

set foragingbudget 0

set socialbudget 0

set day day + 1

set dailypathlength 0

dailyactivity

end

to import-network

set-default-shape nodes "monkey"

import-attributes

import-links

end

;; This procedure reads in a files that contains node-specific attributes

;; including an unique identification number

to import-attributes

;; use CAREFULLY to ensure the file is

;; closed if there is an error and to notify

;; user of the error

carefully [

;; This opens the file, so we can use it.

file-open "attributes.txt"

;; Read in all the data in the file

;; data on the line is in this order:

;; node-id attribute1 attribute2

while [not file-at-end?]

[

;; this reads a single line into a three-item list

let items read-from-string (word "[" file-read-line "]")

create-custom-nodes 1 [

set node-id item 0 items

set weight item 1 items

set dailyproteinrequirement	item 2 items

set dailyenergyrequirement	item 3 items

set proteinintakerate	item 4 items

set energyintakerate	item 5 items

set waterintakeneeded	item 6 items

set socialtimeneeded	item 7 items

set restingtimeneeded	item 8 items

set category item 9 items

]

]

]

;; this is the error handling block of the carefully command

[

user-message (word "Error reading attributes.txt: " error-message)

]

file-close

end

;; This procedure reads in a file that contains all the links

;; The file is simply 3 columns separated by spaces. In this

;; example, the links are directed. The first column contains

;; the node-id of the node originating the link. The second

;; column the node-id of the node on the other end of the link.

;; The third column is the strength of the link.

to import-links

carefully

[

;; This opens the file, so we can use it.

file-open "links.txt"

;; Read in all the data in the file

while [not file-at-end?]

[

;; this reads a single line into a three-item list

let items read-from-string (word "[" file-read-line "]")

ask get-node (item 0 items)

[

__create-link-to get-node (item 1 items)

[ set label item 2 items ]

]

]

]

;; this is the error handling block of the carefully command

[

user-message (word "Error reading links.txt: " error-message)

]

file-close

end

;; Helper procedure for looking up a node by node-id.

to-report get-node [id]

report one-of nodes with [node-id = id]

end
